# Neurological Manifestation of Neonatal Acute Kidney Injury: Focusing on the Clinico-Radiological Profile

**DOI:** 10.7759/cureus.69253

**Published:** 2024-09-12

**Authors:** Purnima Gupta, Ankit Kumar Meena, Esha Parakh, Arvinder Wander, Bhanupratap Rathore, Hemant Jangid, Manish Parakh

**Affiliations:** 1 Department of Pediatrics, Dr. Sampurnanand Medical College, Jodhpur, IND; 2 Department of Pediatrics, All India Institute of Medical Sciences, Bathinda, IND; 3 School of Medicine, Aston Medical School, Aston University, Birmingham, GBR; 4 Department of Radiology, Dr. Sampurnanand Medical College, Jodhpur, IND

**Keywords:** cerebral sinovenous thrombosis, creatinine, kdigo, neonatal acute kidney injury, serum urea

## Abstract

Purpose

This study aimed at studying the neurological manifestation of neonatal acute kidney injury, focusing on the clinico-radiological profile.

Methodology

In this cross-sectional study, newborns hospitalized in the neonatal intensive care unit of a tertiary care hospital were enrolled over a study period of one year. As per the Kidney Disease: Improving Global Outcome (KDIGO) criteria, 74 neonates were enrolled, and magnetic resonance imaging (MRI) was performed on the same neonates.

Result

In this study, acute kidney injury (AKI) was seen more often in neonates with admission weights between 1,500 and 2,499 grams, accounting for 52.7% of total study participants. In the current study, neonates admitted with AKI presented more with signs and symptoms of encephalopathy, such as lethargy (78.4%), seizures (64.8%), and irritability (35.1%) at admission. Signs and symptoms of fever and decreased urine output were more common after the first week of life. Abnormal MRI findings were observed in 64.9% of neonates with AKI. The mean blood urea and serum creatinine levels in neonates with abnormal MRI were 188.14 ± 108.25 mg/dL and 2.93 ± 2.16 mg/dL, respectively. The mean blood urea and serum creatinine levels in neonates with normal MRI were 169.84 ± 65.45 mg/dL and 2.41 ± 1.85 mg/dL, respectively. Of the 74 neonates enrolled with AKI, 12 (16.21%) had CSVT. These neonates had a mean blood urea level of 231.58 ± 111.66 mg/dL (p = 0.047) and a mean creatinine level of 3.77 ± 2.78 mg/dL.

Conclusion

Neonatal AKI has a variable presentation with high mortality and morbidity. Elevated serum urea and creatinine can be used to predict CSVT.

## Introduction

Acute kidney injury (AKI) is characterized by a rapid decline in the renal capacity to maintain water and electrolyte homeostasis and a decrease in the glomerular filtration rate. If the mother has normal kidney function, AKI in term neonates refers to a gradual increase in plasma creatinine by more than 1.5 mg/dL over at least 24-48 hours. Between 8% and 24% of neonatal intensive care unit (NICU) patients have been found to have AKI [1-7]. Early consequences of AKI in newborns include mortality and prolonged hospitalization due to diverse illnesses such as sepsis, convulsions, and uremic encephalopathy. AKI is one of the most significant disorders among NICU patients due to its high incidence rate (8-24%) in hospitalized neonates and its high mortality rate (20-50%) [3,4,6-9].

Both acute and chronic renal disorders can be dangerous to the central nervous system (CNS) [10]. Following AKI, osmolality disruption, inflammation, and retention of nitrogenous end products (uremic toxins) are potential contributory factors for brain involvement. Urea, creatinine, guanidine, and homocysteine are a few examples of the uremic retention products produced due to a decline in renal function to remove nitrogenous waste products and their ongoing production. Numerous toxins interfere with cellular function, leading to neurotoxicity and endothelial vascular damage. The blood-brain barrier (BBB) and brain transporters are damaged, and high serum sodium levels and elevated plasma osmolality in the brain encourage the generation of reactive oxygen species.

Additionally, immunological responses following AKI result in cytokine-induced changes in BBB permeability, setting off inflammatory cascades and subsequent inflammation-related brain injury. The kidneys play crucial roles in cerebral homeostasis, including excreting toxins and medications, adjusting neurotransmitter and cytokine concentrations, and regulating the acid-base and water-electrolyte balance. Unfortunately, the link between AKI and brain problems has received little study.

Following kidney damage, uremic encephalopathy develops more rapidly in acute injury than in chronic renal disease [11,12]. Because patients with AKI have less time to adjust to uremia than those with chronic renal disease, uremic encephalopathy is more common and has more severe consequences [13].

## Materials and methods

In this cross-sectional study, newborns hospitalized in the NICU of a tertiary care hospital were enrolled over a study period of one year. Neonates enrolled in this study fulfilled the AKI definition as per Kidney Disease: Improving Global Outcome (KDIGO) criteria. Stage 1 AKI is defined as a rise in serum creatinine ≥0.3 mg/dL in 48 hours from the baseline value or an increase in serum creatinine by ≥1.5-1.9 times the baseline serum creatinine. Stage 2 AKI is defined as an increase in serum creatinine ≥2-2.9 times the baseline serum creatinine value, and Stage 3 AKI is defined as a rise in serum creatinine of at least 2.5 mg/dL or an increase in serum creatinine by ≥3 times the baseline serum creatinine value [14]. This study did not include urine output criteria because it is often challenging to measure urine output in infants, and nonoliguric AKI is quite common in this population. Baseline serum creatinine was defined as the lowest previous serum creatinine value.

Methods of estimation of serum creatinine and blood urea nitrogen

The institute's central biochemistry laboratory measured serum creatinine using a fully automated analyzer (ERBA EM360, Germany). It uses the alkaline picrate (Jaffe’s) method [15] to estimate serum creatinine using commercially available kits. Blood urea was estimated using the urease hypochlorite method with Autozyme urea reagent and read on the automatic (ERBA EM360, Germany) machine using spectrophotometry principles.

Neuroimaging protocol

After stabilization, a magnetic resonance imaging (MRI) brain scan with time of flight magnetic resonance venogram (MRV) was performed on all neonates. Neonates were evaluated using a 1.5 Tesla MRI system (Philips, Achieva, Amsterdam). The neonates were supine, and their heads were securely placed in the receiver coil. The scan was performed under the supervision of a qualified radiologist posted in the workstation. The following sequences were performed on all patients: T2 axial (repetition time/echo time (TR/TE) 3,600 ms/100 ms, slice thickness 5 mm, slices 20, field of view (FOV) 240 mm), T2 coronal (TR/TE 3,500 ms/116 ms, slice thickness 3 mm, slices 18, FOV 240 mm), fluid-attenuated inversion recovery (FLAIR) axial (TR/TE 11,000 ms/140 ms, slice thickness 5 mm, slices 19, FOV 240 mm), diffusion-weighted imaging (DWI) and apparent diffusion coefficient (ADC) map (TR/TE 2,319 ms/95 ms), gradient echo sequence (TR/TE 504 ms/23 ms, slice thickness 5 mm, FOV 240 mm, duty factor (DF) 30%, flip angle 20 degrees), T1 axial (TR/TE 488 ms/15 ms, slice thickness 5 mm, slices 19, FOV 240 mm), MRV (TR/TE 21 ms/9.9 ms, slice thickness 0.9 mm, slices 10, FOV 230 mm). The MRI brain scan systematically evaluated the following brain structures: ventricles, corpus callosum, gray matter, white matter, limbic system, basal ganglia, brain stem, cerebellum, internal capsule, and cranial vault. A panel of experts in radiodiagnosis reviewed all reports.

Statistical analysis

A semi-structured Proforma was used to enter the particulars of the study subjects. Statistical analysis was performed using IBM SPSS Statistics for Windows, Version 24 (Released 2016; IBM Corp., Armonk, New York). Continuous variables were presented as the mean and standard deviation, and categorical variables were presented as numbers and percentages. To compare means, a T-test and ANOVA were used. To evaluate sensitivity and specificity, a chi-square test was carried out to determine correlations between categorical factors and acute renal injury, and a receiver operating characteristic (ROC) curve was used to evaluate sensitivity, specificity, and positive predictive value (PPV) and negative predictive value (NPV).

Sample size

The sample size was calculated at a 95% confidence interval to verify an expected 22% proportion of brain lesions in AKI.

n = (Z_1-a/2_)^2 ^× p × (100 - p)/E^2^

n = (1.96)^2 ^× 22 × (100 - 78)/(10)^2^

where Z_1-a/2 _is the standard normal deviation for a 95% confidence interval (taken as 1.96), p is the expected proportion of brain lesions in AKI, and E is the absolute allowable error (taken as 10%). The sample size was calculated to be minimum 68 subjects.

## Results

A total of 422 neonates were screened, and 74 were diagnosed with AKI as per the KDIGO definition. MRI was performed on all 74 neonates who fulfilled the predefined inclusion criteria. Of these 74 study participants, 44 (59.4%) were male, and 30 (40.5%) were female. The mean age of presentation in males (6.79 ± 5.95 days) was earlier than that in female newborns (9.96 ± 7.18 days; p = 0.042). AKI was seen more often in neonates with admission weights between 1,500 and 2,499 grams, accounting for 52.7% of study participants. The mean weight on admission for this cohort was 2,288.24 ± 534.97 grams. In the current study, there was no significant difference in weight on admission between male and female neonates (p = 0.103). Seven neonates (9.46%) had preeclamptic mothers during their pregnancy. Nineteen out of 74 (25.7%) neonates with AKI had a history of perinatal asphyxia at birth, and 27 out of 74 (36.4%) had a history of being improperly fed, such as top feed or improperly diluted formula feed (Table 1).

**Table 1:** Baseline Characteristics of Study Participants The mean between the two independent groups was compared with the t-test. A p-value of ≤0.05 was considered statistically significant.

Variables	Category	N = 74	P-value
Gestation	Term	91.9% (68)	-
	Preterm	8.1% (6)	-
Gender	Male	59.4% (44)	-
	Female	40.5% (30)	-
Mean gestational age (weeks)	-	38.10 ± 1.54	-
The mean age of onset of symptoms (days)	Male	6.79 ± 5.95 days (44)	0.042
	Female	9.96 ± 7.18 days (30)	
The mean age of admission (days)	Male	9.06 ± 7.18 days (44)	0.048
	Female	12.76 ± 8.60 days (30)	
Mean birth weight (grams)	Male	2,713.40 ± 415.35 (44)	0.103
	Female	2,532.50 ± 527.19(30)	
Weight at admission (grams)	<1,500	6.75% (5)	-
	1,500–2,499	52.70% (39)	
	>2,500	40.54% (30)	

In the current study, neonates admitted with AKI presented with more signs and symptoms of encephalopathy, such as lethargy (78.4%), seizures (64.8%), and irritability (35.1%). The second most common clinical feature was fever (70.27%) at admission. Fever and decreased urine output were reported more commonly after the first week of life.

Out of the 74 neonates enrolled in this study, MRI resulted in abnormal findings in 48 (64.9%) neonates with AKI. The mean blood urea and serum creatinine levels in neonates with abnormal MRI were 188.14 ± 108.25 mg/dL and 2.93 ± 2.16 mg/dL, respectively. The mean blood urea and serum creatinine levels in neonates with normal MRI were 169.84 ± 65.45 mg/dL and 2.41 ± 1.85 mg/dL, respectively. This difference between normal and abnormal MRI brain changes with mean blood urea and serum creatinine was statistically insignificant. In the current study, gray matter involvement was seen in many neonates. Superficial gray matter was involved in 37 of 74 (50%) neonates, and deep gray nuclei (basal ganglia and thalami) in 24 (32.43%) neonates. White matter involvement was seen in 23 (31.08%) neonates. Deep gray nucleus involvement was seen with a mean blood urea of 188.87 ± 119.54 mg/dL and a mean serum creatinine of 3.06 ± 2.17 mg/dL. Overall, when lesions in the MRI brain scans were evaluated, the cerebral hemisphere (38 of 74) was shown to be involved in the most significant number of cases, followed by the basal ganglia in 20 out of 74 study participants. No statistically significant correlation was found between the involved brain structures and blood urea (p = 0.661) or serum creatinine levels (p = 0.214) (Table 2).

**Table 2:** Distribution of Lesions in MRI Brain and Their Mean Blood Urea (mg/dL) and Serum Creatinine (mg/dL) Levels The mean between the groups was compared with ANOVA. A P-value ≤0.05 was considered statistically significant.

Distribution of the lesion (N)	Serum creatinine (mg/dL, mean + SD)	Blood urea (mg/dL, mean + SD)
Cerebellum (5)	4.15 ± 3.11	232.60 ± 143.5
Basal ganglia (20)	3.38 ± 2.24	207.65 ± 122.68
Internal capsule (10)	3.97 ± 2.71	210.40 ± 111.69
Cerebral hemisphere (38)	2.70 ± 2.00	178.52 ± 104.63
Thalami (16)	2.76 ± 2.01	173.81 ± 115.28
Brainstem (03)	2.10 ± 1.32	120.0 ± 28.47
Corpus callosum (16)	2.36 ± 1.91	177.50 ± 104.02
	P-value=0.214	P-value=0.661

The possible etiology based on neuroimaging was further characterized as metabolic imbalance secondary to dyselectrolytemia, hypoglycemia (19 of 48), hypoxic-ischemic changes (15 of 48; 19 out of 74 had a history of birth asphyxia), hemorrhage (8 of 48), meningitis (5 of 48), and hyperbilirubinemia (1 of 48).

Out of 74 neonates, 55 had undergone ultrasound sonography (USG) of the cranium before their MRI brain scans. Twenty (27.03%) neonates had abnormal MRI findings despite having normal USG results, demonstrating that relying solely on USG leads to a high chance of missing neurological abnormalities.

Among the 74 neonates, 12 (16.22%) had CSVT that was visible on MRV. Of the 12 neonates with CSVT, 10 (83.3%) had seizures at the time of admission, and 8 (66.7%) were encephalopathic (lethargy, irritability). The mean blood urea level was significantly higher (231.58 ± 111.66 mg/dL) in children with CSVT compared to neonates without CSVT (172.06 ± 89.69 mg/dL; p = 0.047).

**Figure 1:**
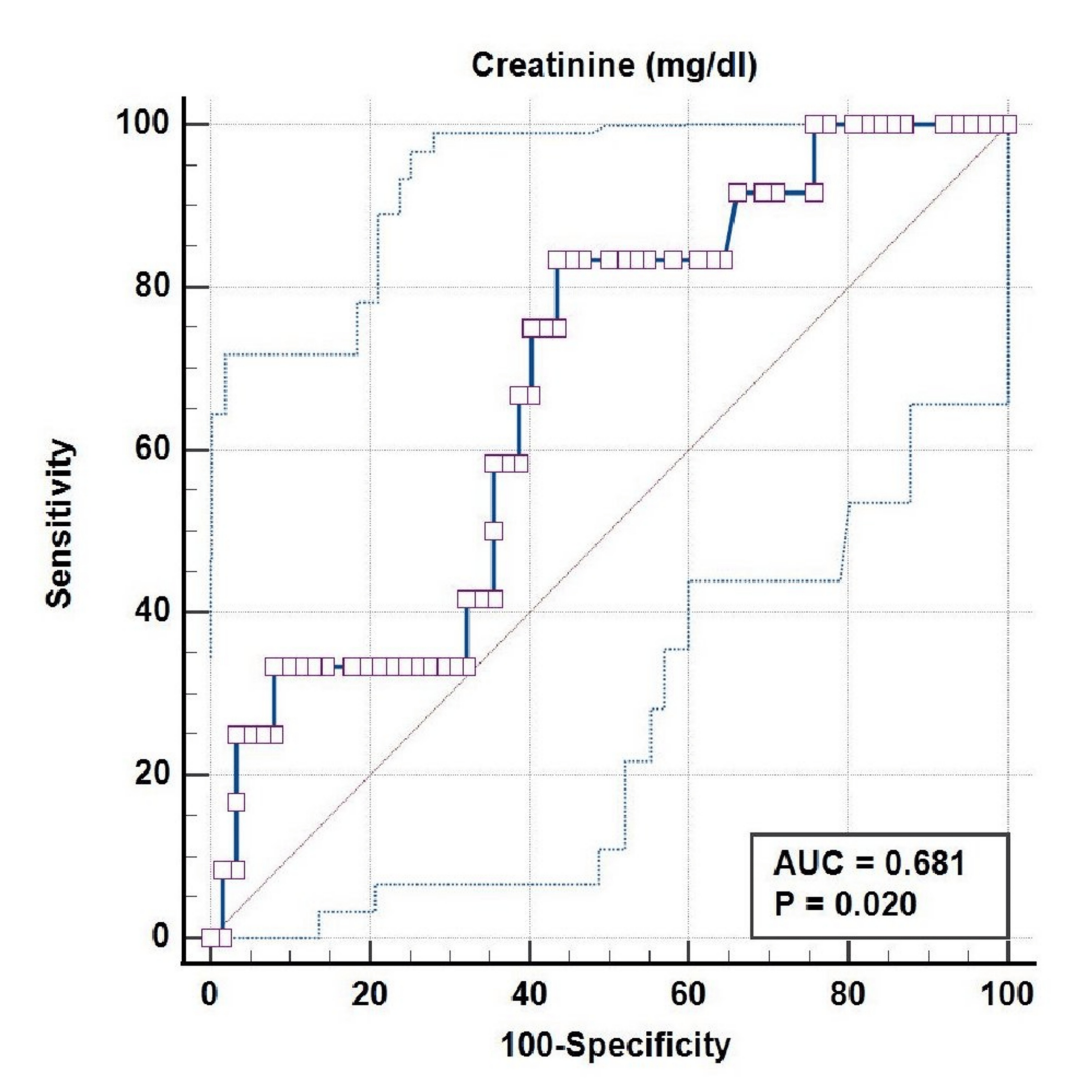
ROC Curve Between CSVT and Serum Creatinine at Admission in AKI Neonates: AUC = 0.681 (95% CI = 0.562-0.784; P = 0.020) ROC: receiver operating characteristic, CSVT: cerebral sinovenous thrombosis, AKI: acute kidney injury, AUC: area under the curve, CI: confidence interval.

The ROC curve plotted between CSVT and serum creatinine at admission in AKI neonates showed an area under the curve of 0.681 (95% CI = 0.562-0.784), with statistical significance (p = 0.020). Using a serum creatinine level cut-off of >1.85 mg/dL for the presence of CSVT yielded sensitivity, specificity, PPV, and NPV of 83.33%, 56.45%, 27.00%, and 94.60%, respectively, in the ROC curve (Figure 1).

**Figure 2:**
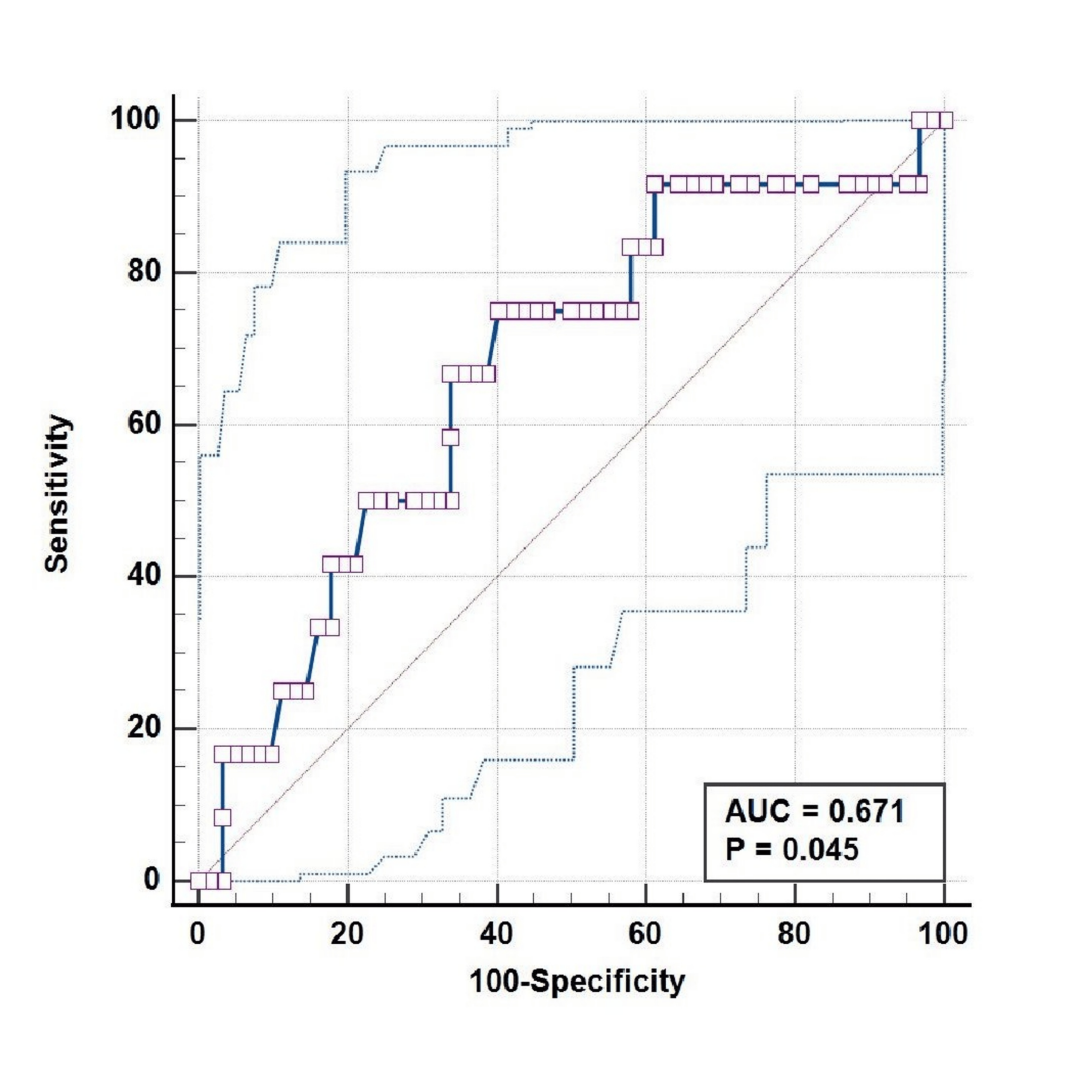
ROC Curve Between CSVT and Blood Urea (mg/dL) at Admission in AKI Neonates: AUC = 0.671 (95% CI = 0.552-0.776; P = 0.045) ROC: receiver operating characteristic, CSVT: cerebral sinovenous thrombosis, AKI: acute kidney injury, AUC: area under the curve, CI: confidence interval.

The ROC curve plotted between CSVT and blood urea at admission in neonates with AKI showed an area under the curve of 0.671 (95% CI = 0.552-0.776), with statistical significance (p = 0.045). Using a blood urea cut-off of > 159 mg/dL for CSVT yielded sensitivity, specificity, PPV, and NPV of 75%, 59.68%, 26.50%, and 92.50%, respectively (Figure 2).

**Figure 3:**
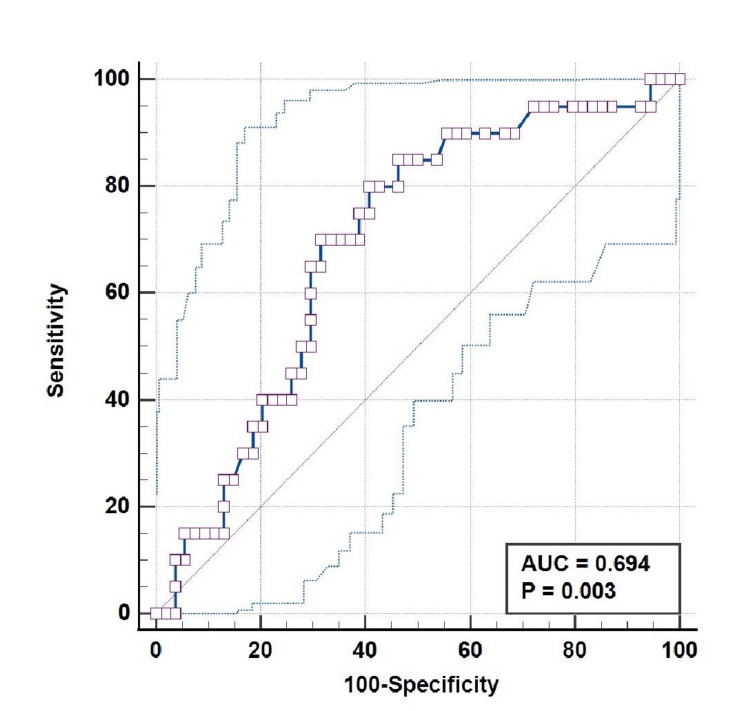
ROC Curve Between Serum Creatinine (mg/dL) at the Time of Admission and Basal Ganglia Involvement in the Brain: AUC = 0.694 (95% CI = 0.562-0.784; P = 0.003) ROC: receiver operating characteristic, AUC: area under the curve, CI: confidence interval.

The ROC curve plotted between basal ganglia involvement and serum creatinine at admission in AKI neonates showed an area under the curve of 0.694 (95% CI = 0.562-0.784), with statistical significance (p = 0.003). Using a serum creatinine level cut-off of >1.79 mg/dL resulted in a sensitivity of 80%, specificity of 59.26%, PPV of 42.10%, and NPV of 88.90% (Figure 3).

## Discussion

In the current study, most neonates diagnosed with AKI were term (91.89%), with the rest being preterm. A higher incidence of AKI in term neonates can be attributed to comorbidities such as perinatal asphyxia, septicemia, and meconium aspiration syndrome. The incidence of AKI was observed to be lower in preterm neonates; this may be due to the well-established fact that preterm neonates are referred early and hospitalized for concerns related to prematurity. Additionally, more robust feeding monitoring is provided for preterm neonates.

In this study, most neonates were admitted with a mean weight of 2,288.24 ± 534.97 grams. AKI was seen more often in neonates with admission weights between 1,500 and 2,499 grams, accounting for 52.7%. There was no significant difference in the mean weight of male and female neonates at admission.

AKI was most commonly observed in the first few days of life; similarly, in the present study, most neonates developed AKI between the sixth and tenth days of life, with a mean age of admission of 8.08 ± 6.62 days. In a study by Ali et al. [16], the mean age of admission was 5.73 ± 7.20 days for males and 6.77 ± 6.16 days for females. In the present study, the mean age of admission for females was also higher than for males. The delay in presentation for female neonates may be due to gender-based discrimination practiced in the community.

The gender distribution reported in various studies around the world has documented a higher prevalence of AKI in males compared to females. In the present study, out of 74 neonates, there were 44 (59.45%) males and 30 (40.54%) females, which was statistically significant and comparable to observations by Halder et al. [17] and Gharehbaghi et al. [18].

The expected non-pathological weight loss in the first 10 days of life is 1% daily. In the present study, 97.3% of neonates with AKI had a weight loss of ≥10% of birth weight at the time of admission. This weight loss exceeded the maximum expected daily physiological weight loss. Neonates delivered in resource-limited settings, where pediatricians and lactation counselors are not available, are often subjected to inadequate postnatal care and early discharge. Parents and caregivers are often not sensitized to detect clinical features of dehydration at an early stage. Such neonates are admitted when there is a high-grade fever or when they have not passed urine for a long time. This could explain why significant weight loss was noted in the present study.

In the study conducted by Pandya et al. [19], the most common clinical feature noted in neonates with AKI was lethargy (70%), followed by fever (53.3%), significant weight loss, jaundice, and dehydration. In the present study, the majority of babies had lethargy (78.38%) as their chief complaint at the time of presentation, followed by fever (70.27%); a significant proportion (64.86%) of neonates also presented with seizures. Boskabadi et al. [20] demonstrated in their study that the main complaints for admission were fever (50%), lethargy (45.3%), jaundice (39.6%), irritability (26.4%), seizures (22.6%), and excessive weight loss (7.5%).

Literature on the radiological findings of brain injury in neonates with AKI is minimal. In the present study, 48 (64.86%) neonates had abnormal MRI findings, while 26 had normal MRI brain findings. It was noted that neonates with abnormal MRI findings had a mean blood urea value of 188.14 ± 108.25 mg/dL and a mean serum creatinine value of 2.93 ± 2.16 mg/dL. The mean blood urea and serum creatinine values recorded in neonates with normal MRI were 169.84 ± 65.45 mg/dL and 2.41 ± 1.85 mg/dL, respectively.

A retrospective study by Kim et al. [21] included 10 patients (nine men and one woman; mean age, 58 years; age range, 17-76 years) with clinically proven uremic encephalopathy and abnormalities on brain imaging. In nine of 10 patients with chronic renal failure, MRI showed bilateral, expansile, and symmetric basal ganglia lesions with increased signal intensity on T2-weighted images (T2WI), FLAIR, and ADC maps, compatible with vasogenic edema. Similarly, in the current study, 27% of neonates with AKI had basal ganglia involvement.

The ROC curve plotted between basal ganglia involvement and serum creatinine at admission in AKI neonates revealed an area under the curve of 0.694 (95% CI = 0.562-0.784; p = 0.003) using a serum creatinine level of >1.79 mg/dL, with a sensitivity of 80%, specificity of 59.26%, PPV of 42.10%, and NPV of 88.90%.

The reported incidence of neonatal thrombosis varies from 6.9 to 15 per 1,000 neonates admitted to the NICU [22-25]. In the present study, 16.21% of neonates were observed to have CSVT. These neonates had a mean blood urea level of 231.58 ± 111.66 mg/dL (p = 0.047) and a mean creatinine level of 3.77 ± 2.78 mg/dL. To the best of our knowledge, this is the first study to examine the incidence of CSVT in neonates with AKI; however, the available data are very limited.

The most common clinical manifestations of perinatal venous thrombosis cited in the literature include hypotonia, lethargy, poor feeding or respiratory distress, seizures, and changes in eyeball movements. In the current study, neonates presented with various features of encephalopathy, the most common being seizures in 10 (83.33%) out of 12 neonates, lethargy in 8 (66.67%), and hyperreflexia in 6 (50%). Fitzgerald et al. [26] found that 24 (57%) of the 42 patients in their study presented with seizures and apnea or respiratory distress, poor feeding, lethargy, and hypotonia. Twenty-eight (67%) of the 42 neonates in their study had multiple symptoms.

The strength of our study is that it is the first to examine the incidence of CSVT in neonates with AKI, as it may be entirely missed due to similar clinical presentations (lethargy, poor feeding, seizures) and requires a different treatment approach. However, all study participants were extramural to the NICU, so we could not conclude the exact reason for AKI. Hypoxic-ischemic insult is a significant cause of AKI in developing countries and may overlap with the neuroimaging features of neonatal AKI.

## Conclusions

Neonatal AKI has a variable presentation with high mortality and morbidity. Elevated serum urea and creatinine levels can be used to predict CSVT. However, a study with a larger sample size is needed to generalize this observation. While serum urea and creatinine may indicate basal ganglia involvement, they are not specific to other brain regions. A longitudinal study will help determine the neurodevelopmental outcomes.
